# MicroRNAs of *Toxocara canis* and their predicted functional roles

**DOI:** 10.1186/s13071-016-1508-3

**Published:** 2016-04-23

**Authors:** Guangxu Ma, Yongfang Luo, Honghong Zhu, Yongli Luo, Pasi K. Korhonen, Neil D. Young, Robin B. Gasser, Rongqiong Zhou

**Affiliations:** Department of Veterinary Medicine, Rongchang Campus of Southwest University, Chongqing, 402460 The People’s Republic of China; Faculty of Veterinary and Agricultural Sciences, The University of Melbourne, Parkville, Victoria 3010 Australia

**Keywords:** *Toxocara canis*, microRNAs (miRNAs), Reproduction and development, Host-parasite interactions, Drug resistance

## Abstract

**Background:**

*Toxocara canis* is the causative agent of toxocariasis of humans and other animals. This parasitic nematode (roundworm) has a complex life cycle, in which substantial developmental changes and switches occur. As small non-coding RNAs (sRNAs) are key regulators of gene expression in a wide range of organisms, we explored these RNAs in *T. canis* to provide a basis for future studies of its developmental biology as well as host interactions and disease at the molecular level.

**Methods:**

We conducted high-throughput RNA sequencing and bioinformatic analyses to define sRNAs in individual male and female adults of *T. canis*.

**Results:**

Apart from snRNA and snoRNA, 560 and 619 microRNAs (miRNAs), including 5 and 2 novel miRNAs, were identified in male and female worms, respectively, without piRNAs being detected in either sex. An analysis of transcriptional profiles showed that, of 564 miRNAs predicted as being differentially transcribed between male and female individuals of *T. canis*, 218 miRNAs were transcribed exclusively in male and 277 in female worms. Functional enrichment analysis predicted that both male and female miRNAs were mainly involved in regulating embryonic morphogenesis, hemidesmosome assembly and genetic information processing. The miRNAs differentially transcribed between the sexes were predicted to be associated with sex determination, embryonic morphogenesis and nematode larval development. The roles of miRNAs were predicted based on gene ontology (GO) and KEGG pathway annotations. The miRNAs *Tc*-miR-2305 and *Tc*-miR-6090 are proposed to have roles in reproduction, embryo development and larval development, and *Tc*-let-7-5p, *Tc*-miR-34 and *Tc*-miR-100 appear to be involved in host-parasite interactions. Together with published information from previous studies, some miRNAs (such as *Tc*-miR-2861, *Tc*-miR-2881 and *Tc*-miR-5126) are predicted to represent drug targets and/or associated with drug resistance.

**Conclusions:**

This is the first exploration of miRNAs in *T. canis*, which could provide a basis for fundamental investigations of the developmental biology of the parasite, parasite-host interactions and toxocariasis as well as applied areas, such as the diagnosis of infection/disease, drug target discovery and drug resistance detection.

**Electronic supplementary material:**

The online version of this article (doi:10.1186/s13071-016-1508-3) contains supplementary material, which is available to authorized users.

## Background

*Toxocara canis* is an important intestinal nematode of dogs and the principal causative agent of human toxocariasis worldwide [1, 2]. Humans, particularly children [3, 4], can be infected through the accidental ingestion of embryonated eggs of *Toxocara* or infective larvae in raw/undercooked meats or viscera [5–8]. Once the human host is infected, larvae can invade multiple tissues or organs, causing visceral larva migrans, ocular larva migrans, neurotoxocariasis and/or covert toxocariasis [7, 9, 10]. Although many infections are likely to be asymptomatic, the relationship between toxocariasis and epilepsy as well as asthma has raised considerable public concern [11–13]. In addition, epidemiological studies have indicated a relatively high prevalence (12–93 %) of toxocariasis in humans in some African, Asian and Latin American countries [1, 3, 4, 14–16], although prevalence levels are likely to be underestimated due to limited investigations around the world [17].

Various studies have given improved insights into the epidemiology of *Toxocara* species using molecular methods [18], and knowledge of the genome and transcriptomes is now helping us gain a better understanding of the fundamental molecular biology, biochemistry and physiology of *T. canis* [19, 20]. Central to many biological processes of this parasite is knowledge of the regulators of survival, development, reproduction, invasion and immune evasion, namely small RNAs (sRNAs). However, there is no information on these RNAs for any ascaridoid nematode, except *Ascaris* [21].

The sRNAs are usually 20–30 nucleotides (nt) in length, and are key players in gene perturbation by interacting with mRNAs (e.g., microRNAs (miRNAs) and silencing RNAs), and by regulating genes through chromatin modification (e.g., small interfering RNAs and piwi-interacting RNAs, piRNAs) (cf. [22–25]). Specifically, since lin-4 was originally identified in *Caenorhabditis elegans* [26], knowledge of miRNAs in organisms ranging from unicellular to multicellular organisms has expanded substantially in the last decade [27], thereby significantly broadening our knowledge of the roles of miRNAs in regulating biological processes [28–30]. Recently, some studies of parasites have elucidated the regulatory roles of some miRNAs in survival [31, 32], tissue development and reproduction [32–34]. Of particular interest is that selected miRNAs of parasitic nematodes might also play roles in the regulation of parasite-host interactions [35–37] and drug resistance [29, 38]. Given the lack of information for most ascaridoid nematodes, we characterized here the sRNAs of *T. canis* and conducted comparative analyses to provide a basis for future fundamental investigations of development, reproduction, host-parasite interactions, and possibly for applied areas, such as the diagnosis of infection and detection of drug resistance as well as drug target discovery.

## Methods

### Ethics statement

All experiments involving dogs were approved by Southwest University, China, using a protocol that complied with the requirements of the Ethics Procedures and Guidelines of the People’s Republic of China.

### Procurement of adult *T. canis*

Adult *T. canis* were expelled from naturally infected dogs in the Rongchang Campus Animal Hospital of Southwest University, China. Male and female adult worms of *T. canis* were washed three times in sterile physiological saline (37 °C), and each worm was snap-frozen separately and stored at −80 °C until use. The specific identity of each worm was verified by morphological and molecular identification using established descriptions and methods [39, 40].

### Small RNA library construction and RNA-sequencing

High-quality total RNA was extracted (separately) from the entire body of a male and a female adult worm using Trizol reagent (Invitrogen, Carlsbad, CA, USA). RNA yield and quality were measured spectrophotometrically (BioPhotometer, Eppendorf, Germany). The total RNA (20 μg) from each of the two samples was fractionated using Novex 6 % TBE-Urea gels (Invitrogen, Carlsbad, CA, USA), and the fragments of 18–30 nt were ligated with 5′ and 3′ adaptors (Illumina) for reverse transcription. The resultant first-strand cDNA was amplified with a small RNA primer set to enrich the libraries, and the cDNA libraries were sequenced (BGI-Shenzhen, China) using Illumina technology (HiSeq2000; sequencing length: 50 nt; paired-end).

### Processing and analysis of sequencing data

The raw sequence reads were pre-processed for quality, and adaptors, reads of < 18 nt and low-complexity reads were removed; then, the length distribution of the “clean” reads was assessed. All clean reads were mapped to the transcriptome of *T. canis* (accession no. GSE75536) using the program SOAP [41]. The identification and annotation of matched sRNAs were conducted by homology-based searching against ribosomal RNA (rRNA), small nucleolar RNA (snoRNA), small nuclear RNA (snRNA), piRNA and transfer RNA (tRNA) data in GenBank within the National Center for Biotechnology Information (NCBI; [42]) and Rfam release 10.1 [43] databases using BLASTn. Perfectly matched sequences were excluded, and the unmatched sequences were compared with the precursor/mature miRNAs in miRBase release 18.0 [44] to identify known miRNAs, allowing two mismatches and no gaps. The miRNAs levels (both strands) were estimated based on read counts; the minimum number of counts was set at 100. Novel miRNAs were predicted based on their secondary structures, the Dicer cleavage site and the minimum free energy (−18 kcal/mol) using the tool MIREAP [45]. Transcriptomic and small RNA data have been deposited in the NCBI Gene Expression Omnibus (http://www.ncbi.nlm.nih.gov/geo) under accession nos. GSE75536 and GSE68710, respectively.

### The prediction of miRNA targets and annotation

Target genes were predicted using RNAhybrid software [46], and their functions were annotated using gene ontology (GO) [47] and KEGG pathway analyses [48]. Functional enrichment analysis of miRNA targets was conducted. The significance of GO and pathway enrichment was set at a *P*-value of ≤ 0.05 (corrected). In addition, miRNAs targeting genes linked to reproductive processes, host-parasite interactions or drug resistance were predicted on the basis of enriched GO terms and/or pathways.

### Comparative analysis between the worms

The transcriptional profiles for identified miRNAs were clustered using tag2miRNA software (custom-designed by BGI-Shenzhen), and the transcription levels of novel miRNAs were produced by summing up the counts of miRNAs with no more than 3 mismatches at the 5′ and 3′ ends, and with no mismatch in the middle of their sequence alignment. To infer a list of miRNAs that were exclusive to male and female libraries, the fold-change was calculated (log_2_ (female/male)) from the normalised transcription (miRNA count/total count of clean reads × 1,000,000), in which a given value (0.01) was added to that of extremely lowly transcribed miRNAs. The miRNAs differentially transcribed between male and female *T. canis* were inferred based on a log_2_ fold-change of ≥ 2 in read count using the program EdgeR (http://bioconductor.org/packages/release/bioc/html/edgeR.html). Then, a functional enrichment analysis of differentially transcribed miRNA targets was carried out to predict gender-enriched GO terms and pathways. In addition, miRNAs with gender-enriched annotations and predicted to be involved in reproduction or larval development were identified. Moreover, miRNAs in *T. canis* inferred to be involved in parasite-host interactions were identified by sequence comparison with secretory miRNAs reported previously for some filarioid nematodes, including *Brugia malayi*, *Dirofilaria immitis*, *Loa loa*, *Onchocerca ochengi*, and the strongylid nematode *Heligmosomoides polygyrus* (*bakeri*) [36, 49–51], as were those predicted to play roles in regulating arrested development (dauer) employing larval miRNAs for *Ascaris suum*, *B. malayi, C. elegans* and *H. polygyrus * (*bakeri*) [32, 36, 51, 52]; miRNA matches were defined on the basis of 100 % identity in the seed sequence. The miRNAs sequences were aligned using Clustal software [53], and alignments adjusted manually. Additional analyses and data preparation were conducted in a Microsoft Excel 2013 using standard commands.

### Quantitative real-time PCR (qPCR) assays

To investigate the transcriptional profiles of miRNAs predicted to be involved in developmental and reproductive processes, qPCR was employed to assess levels of transcription for selected miRNAs in the different body parts of the two (male and female) adults of *T. canis*. Total sRNAs were extracted separately from the reproductive tracts, intestines and body walls of the male and female worms using the EasyPure miRNA Kit (TransGen Biotech, Beijing, China). Then, sRNAs were polyadenylated and transcribed into first-strand cDNA, according to the manufacturer’s protocol. To estimate transcription levels, a two-step qPCR was carried out using a *TransScript* Top Green qPCR Supermix (TransGen Biotech, Beijing, China) employing the following cycling protocol: 94 °C/30 s, followed by 40 cycles of 94 °C/5 s and 60 °C/30 s. The primers used are shown in Additional file 1: Table S1. Although no universally applicable normaliser gene has yet been identified [54], based on an appraisal of some previous studies [55–57], we elected to employ the small subunit of the nuclear ribosomal RNA (18S) gene as an internal reference control. Three independent replicates were performed, and the relative transcription level was established using the 2^-ΔCt^ method [58], and presented as $\overline{\mathrm{x}}$ ± standard deviation (SD).

## Results

### Features of sRNA libraries

Libraries were constructed separately for the male and female individuals of *T. canis*, and paired-end sequenced. In total, 11,824,662 and 11,486,831 raw reads were produced by deep sequencing, with high quality tags constituting 99.5 % and 99.4 % of all reads, respectively, from which 11,632,676 and 10,723,433 “clean” reads were derived, following the removal of adaptors, short reads (<18 nt) and low complexity reads. The male sRNAs were between 18 and 28 nt in length, compared with 16–29 nt for female sRNAs. Specifically, 5,792,295 (49.79 %) and 6,094,798 (56.84 %) reads mapped to the total RNA data representing male and female *T. canis*, respectively. Following annotation, snoRNA, snRNA and miRNA accounted for 0.01 %, 0.07 % and 24.92 % of all male sRNAs, compared with 0.01 %, 0.04 % and 32.86 % of female sRNAs, respectively (Table 1). Interestingly, no piRNA sequence was detected in either the male or female sRNA library representing *T. canis*.

**Table 1:** Statistics of the small RNAs sequenced from libraries representing male and female adult individuals of *Toxocara canis*

Description	Male library	Female library
Total number of reads (%)	11,824,662 (100)	11,486,831 (100)
High quality reads (%)	11,766,843 (99.51)	11,415,284 (99.38)
Clean reads (%)	11,632,676 (98.38)	10,723,433 (93.35)
Total sRNA reads (%)	11,632,676 (100)	10,723,433 (100)
Mapping to genome (%)	5,792,295 (49.79)	6,094,798 (56.84)
snoRNA reads (%)	988 (0.01)	615 (0.01)
snRNA reads (%)	7659 (0.07)	4815 (0.04)
miRNA reads (%)	2,899,258 (24.92)	3,524,138 (32.86)
Known miRNAs (n)	555	617
Novel miRNAs (n)	5	2

### Transcriptional profiles and functional enrichment

By aligning miRNA precursors with mature miRNA sequences in miRBase, we defined 555 and 617 ‘known’ miRNAs from 2,899,258 male reads and from 3,524,138 female reads, respectively (Table 1). Particular miRNAs, such as *Tc*-miR-51-3p (with 1,571,239 reads) and *Tc*-miR-3070-2-3p (with 1,333,544 reads) were very highly transcribed in the male *T. canis*, compared with *Tc*-miR-279b-3p (with 451,632 reads), *Tc*-miR-71c-5p (with 340,196 reads), *Tc*-miR-71 (with 339,285 reads) and *Tc*-miR-265 (with 231,054 reads) in the female worm (Additional file 1: Table S2). In addition, 5 novel, male miRNAs and 2 novel, female miRNAs were predicted (Table 1); most of these miRNAs had low levels of transcription, except for novel_*Tc*-miR-47, which was represented by 5888 reads in the male library (Additional file 1: Table S2).

**Fig. 1:**
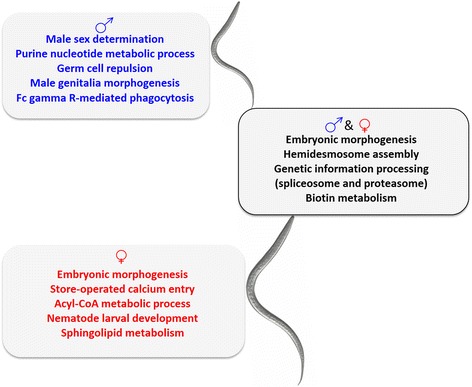
Summary of functional enrichment analyses of microRNAs (miRNAs) in adult worms of *Toxocara canis*. In both male and female worms, miRNAs (i.e. those for which homologs have been described previously for other nematodes) were predicted to be involved in regulating embryonic morphogenesis, hemidesmosome assembly, genetic information processing (including spliceosome and proteasome) and biotin metabolism (right box). Differentially transcribed miRNAs were predicted to be mainly associated with male sex determination, purine nucleotide metabolism process, germ cell repulsion, male genitalia morphogenesis and Fc gamma R-mediated phagocytosis exclusively in the male worm (top box), and with embryonic morphogenesis, store-operated calcium entry, Acyl-CoA metabolic process, nematode larval development and sphingolipid metabolism in the female worm (bottom box)

Target prediction was carried out using transcriptomic data from adult *T. canis*. The results showed that 213 known miRNAs targeted 10,815 genes in the male worm, and 223 known miRNAs targeted 9,076 genes in the female worm, compared with 5 novel miRNAs with 44 target genes in the male *T. canis*, and 2 novel miRNAs with 73 targets in the female *T. canis*. Following GO annotation and pathway enrichment analyses, we inferred that male-enriched miRNAs have roles in regulating the biological processes of embryonic morphogenesis and hemidesmosome assembly, and pathways involving spliceosomes, basal transcription factors, biotin metabolism, proteasome, ribosome, ubiquitin-mediated proteolysis, adherens junction, protein digestion and absorption, focal adhesion and the PI3K-Akt signalling pathway (Fig. 1; Additional file 1: Table S3). On the other hand, female-enriched miRNAs were predicted to be linked to processes including embryonic morphogenesis, RNA polyadenylation, hemidesmosome assembly, regulation of cell division and nucleus organisation (Fig. 1; Additional file 1: Table S3), with pathways associated with spliceosomes, biotin metabolism and proteasome (Additional file 1: Table S3).

### Transcriptional differences between male and female worms, and functional enrichment

**Fig. 2:**
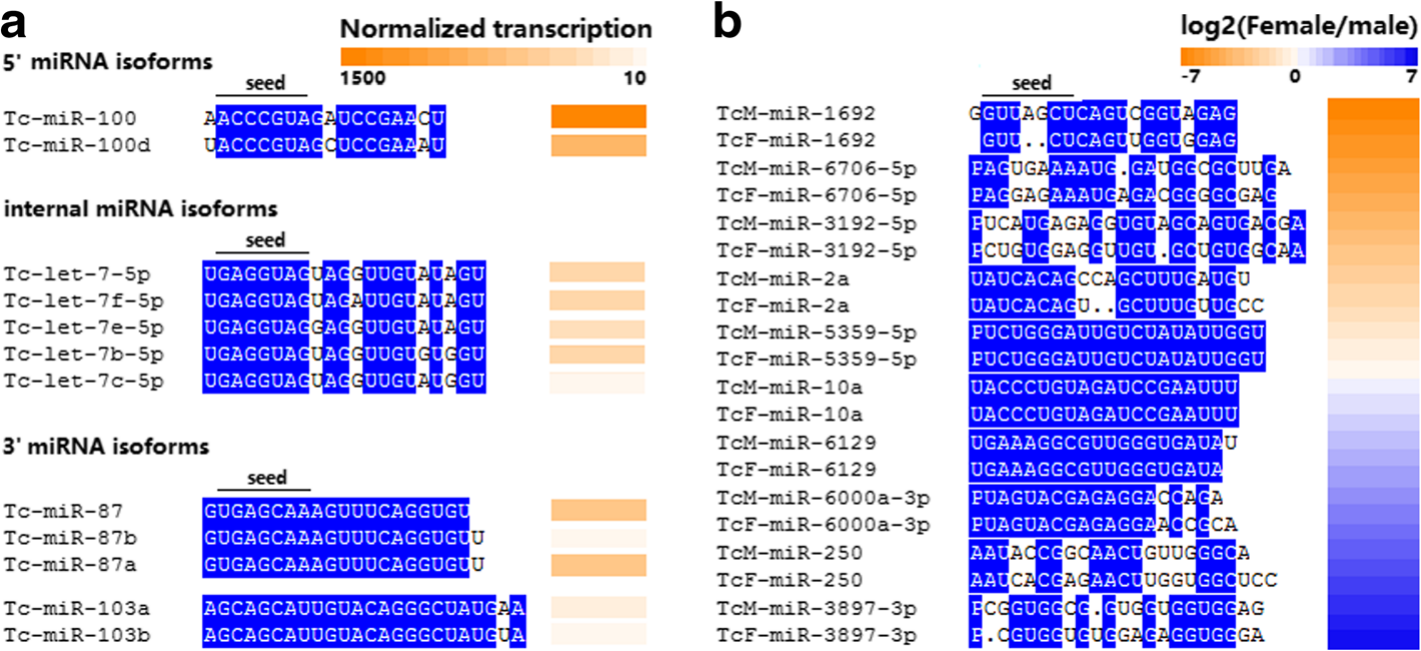
Sequence-dependent transcription profiles for selected microRNAs (miRNAs) in adult *Toxocara canis.* Panel **a**: Normalised transcription for distinct miRNAs *Tc*-miR-100, *Tc*-miR-100d, *Tc*-let-7-5p, *Tc*-let-7b-5p, *Tc*-let-7c-5p, *Tc*-let-7e-5p, *Tc*-let-7f-5p, *Tc*-miR-87, *Tc*-miR-87a, *Tc*-miR-87b, *Tc*-miR-103a and *Tc*-miR-103b. Panel **b**: Log_2_ transcription ratio between the male and female worms for conserved miRNAs, with *Tc*-miR-2a, *Tc*-miR-5359-5p, *Tc*-miR-10a, *Tc*-miR-6129, and *Tc*-miR-6000a-3p having a conserved seed sequences between male and female, and *Tc*-miR-1692, *Tc*-miR-6076-5p, *Tc*-miR-3192-5p, *Tc*-miR-250 and *Tc*-miR-3897 having identical miRNA sequences between the two sexes

In the differential transcription analysis, we predicted 564 gender-biased miRNAs, in terms of read counts and calculated fold-changes (log_2_ female *vs.* male); 218 and 277 of these miRNAs were exclusive to male and female *T. canis*, respectively (Additional file 1: Table S4). In addition, sequence-dependent transcription was recorded in male and in female *T. canis*; for instance, transcription levels differed among miRNAs *Tc*-miR-100, *Tc*-miR-100d, *Tc*-let-7-5p, *Tc*-let-7b-5p, *Tc*-let-7c-5p, *Tc*-let-7e-5p, *Tc*-let-7f-5p, *Tc*-miR-87, *Tc*-miR-87a, *Tc*-miR-87b, *Tc*-miR-103a and *Tc*-miR-103b (Fig. 2a; Additional file 1: Table S5), whereas only limited differences in transcription were recorded for 342 miRNAs shared by male and female *T. canis*, with these miRNAs having a conserved seed sequence between the two sexes (Fig. 2b; Additional file 1: Table S5).

In the functional enrichment analysis, biological processes linked to male-enriched miRNAs in *T. canis* included embryonic morphogenesis, hemidesmosome assembly, masculinization of hermaphroditic germ-line, negative regulation of vulval development, nematode larval development, regulation of DNA-templated transcription, termination, synaptic vesicle priming, embryo development ending in birth or egg hatching, male somatic sex determination, male germ-line sex determination, moulting cycle, collagen and cuticulin-based cuticle, locomotion, purine nucleotide metabolic process, positive regulation of Rac protein signal transduction, positive regulation of Rho protein signal transduction, germ cell repulsion and male genitalia morphogenesis (Additional file 1: Table S6), as well as pathways involving basal transcription factors, spliceosomes and Fc gamma R-mediated phagocytosis (Additional file 1: Table S6). On the other hand, processes linked to female-enriched miRNAs in *T. canis* included hemidesmosome assembly, embryonic morphogenesis, moulting cycle, collagen and cuticulin-based cuticle, positive regulation of smooth muscle contraction, receptor localisation to synapse, regulation of actin cytoskeleton reorganisation, store-operated calcium entry, positive regulation of engulfment of apoptotic cell, short-term memory, barbed-end actin filament capping, acyl-CoA metabolic process and nematode larval development (Additional file 1: Table S6), and enriched pathways including proteasome, sphingolipid metabolism and spliceosomes (Additional file 1: Table S6).

### MiRNAs predicted to be associated with development and reproduction

**Fig. 3:**
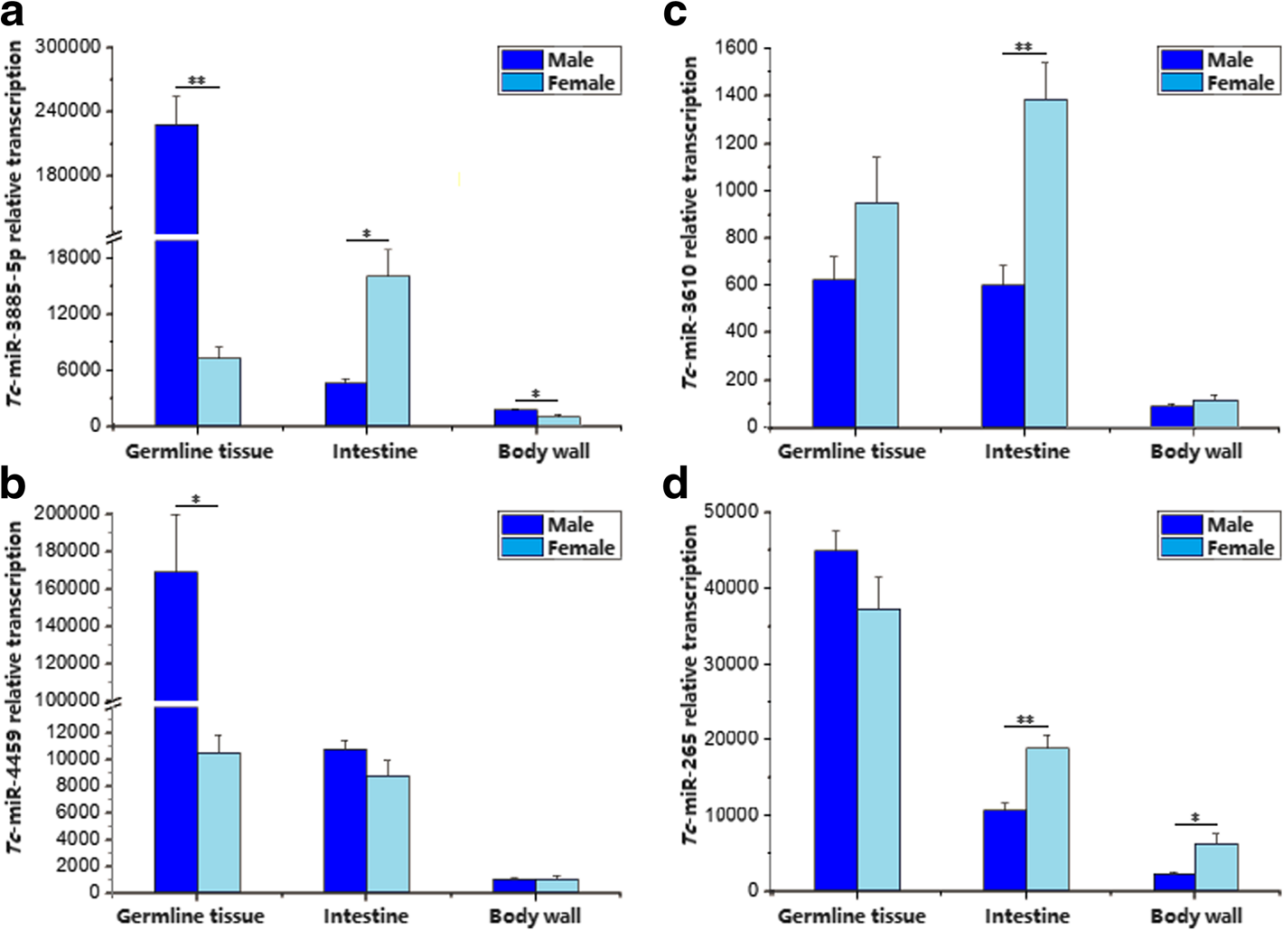
Transcription of microRNAs predicted to be linked to reproductive processes. To estimate the levels of transcription of miRNAs in male and female adult *T. canis*, quantitative real-time PCR was performed employing specific forward and universal reverse primers. The relative levels of *Tc*-miR-3885 (**a**), *Tc*-miR-3610 (**b**), *Tc*-miR-4459 (**c**) and *Tc*-miR-265 (**d**) are indicated (*P = 0.01; **P = 0.001)

Based on GO annotation analysis, we predicted 52 differentially transcribed miRNAs to be involved in developmental and reproductive processes. These miRNAs were classified into 28 seed sequence families (Additional file 1: Table S7). Notably, *Tc*-miR-2305 and *Tc*-miR-6090, with a seed sequence 5′-GGGAGCG-3′ or 5′-GGGGGGC-3′, were inferred to be involved in meiosis, and embryonic and larval development. In addition, qPCR data showed that *Tc*-miR-3885, *Tc*-miR-4459, *Tc*-miR-3610 and *Tc*-miR-265 had different levels of transcription in germline, intestine and body wall between the male and female worms of *T. canis* (Fig. 3). Specifically, *Tc*-miR-3885 (Fig. 3a) and *Tc*-miR-4459 (Fig. 3b) were transcribed significantly higher in the germline tissues in the male than in the female worm, whereas the opposite was the case for *Tc*-miR-3610 (Fig. 3c) and *Tc*-miR-265 (Fig. 3d) in the intestine.

### MiRNAs predicted to be linked to host-parasite interactions

We predicted that 60 miRNAs, including particularly *Tc*-miR-2881, *Tc*-miR-2305 and *Tc*-miR-2861 (Additional file 1: Table S8), have roles in regulating mucin type O-glycan biosynthesis and vesicular transport in *T. canis* and/or modulating immune responses (SNARE interactions in vesicular transport, antigen presentation, intestinal immune network for IgA production, cytokine-cytokine receptor interaction and natural killer cell-mediated cytotoxicity) in the host. In addition, 42 miRNAs, particularly *Tc*-let-7-5p, *Tc*-lin-4, *Tc*-bantam, *Tc*-miR-10, *Tc*-miR-34, *Tc*-miR-71 and *Tc*-miR-100, were predicted to be excreted/secreted by developmental arrested/infective larvae of *T. canis* into host tissues. Moreover, some miRNAs, such as *Tc*-miR-84-5p, *Tc*-miR-34, *Tc*-miR-100, *Tc*-miR-57-5p, *Tc*-miR-125a-5p, *Tc*-miR-71 and *Tc*-miR-753b-3p, with seed sequences including 5′-GAGGUAG-3′, 5′-GGCAGUG-3′, 5′-ACCCGUA-3′, 5′-ACCCUGU-3′, 5′-CCCUGAG-3′, 5′-GAAAGAC-3′ and 5′-GAGAUCA-3′, respectively (Additional file 1: Table S8), were predicted to be involved in host-parasite interactions.

### MiRNAs as putative drug targets or with a possible link to drug resistance

Although no miRNAs were linked to nicotinic acetylcholine receptor-, ligand-gated ion channel- or P-glycoprotein-encoding genes in *T. canis*, some miRNAs (*n* = 35), including *Tc*-miR-2881, *Tc*-miR-5126, *Tc*-miR-2861 and *Tc*-miR-3960, did target ABC transporter, cytochrome P450 and multi-drug resistance-associated protein genes (Additional file 1: Table S9), suggesting an involvement in the regulation of transcription of drug target genes and/or possibly in drug resistance. We also inferred miRNAs (*n* = 83) targeting genes encoding signalling molecules, transcription factors, receptors, kinases and ion channels, suggested to be involved in the basal calcium signalling pathway and the phosphatidylinositol signalling system of *T. canis* (Additional file 1: Table S9).

## Discussion

Genomic, transcriptomic and proteomic investigations are providing important insights into *Toxocara* species as well as parasite-host interactions (cf. [19, 20]). Investigating short non-coding RNAs is part of this focus. Here, we studied miRNA transcription profiles and predicted functions of target genes in individual female and male adults of *T. canis*. More than 60 % of the miRNAs were predicted to be transcribed in a gender-enriched manner in *T. canis*, whereas more than half of these gender-biased miRNAs were conserved on a sequence level and had similar transcription levels. Various mechanisms are associated with the diversification of miRNA sequences, including trimming and tailing [59, 60], which means that various isoforms might display differing transcription levels and be “tailored” to efficiently regulate distinct functions. Apart from the gender-biased transcription predicted, miRNAs differentially transcribed between the two sexes of *T. canis* shared some functional roles, for example, the regulation of embryonic morphogenesis and hemidesmosome assembly, whereas the functional prediction highlighted biological functions for other differentially transcribed miRNAs, particularly in regulating nematode reproduction, male germ-line sex determination, embryonic morphogenesis and larval development.

In the free-living nematode *C. elegans*, miRNAs, such as the originally identified lin-4 and let-7, play key biological roles in regulating development [26, 61]. Although piRNA was not found here in *T. canis*, which was supported by the absence of components of the piRNA pathway from other clade III parasites (e.g., [52, 62, 63]), a considerable number of miRNAs, particularly novel and differentially transcribed representatives, appear to be associated with development and reproduction. Specifically, a number of miRNAs were predicted to be involved in the regulation of embryonic and/or larval development, and miRNAs including *Tc*-miR-265, *Tc*-miR-3885, *Tc*-miR-4459 and *Tc*-miR-3610 were transcribed at higher levels in germline tissues than other body parts of *T. canis*, whereas several miRNAs with a conserved seed sequence, 5′-GGGAGCG-3′ or 5′-GGGGGGC-3′ (e.g., *Tc*-miR-2305 and *Tc*-miR-6090), were inferred to be involved in meiosis. In addition, 342 conserved miRNAs (for which only the seed sequence was conserved) in both male and female adults of *T. canis* might be post-transcriptional regulators in developmental arrested larvae, according to evidence published for *C. elegans*, *Pristionchus pacificus* and *Strongyloides ratti* [32]. Since dauer larvae have been suggested to be a “pre-adaptation” of infective larvae in paratenic animals (cf. [64]), these potential regulators in *T. canis* might play roles in larval development, survival and/or host-parasite interactions.

Helminths secrete miRNAs that are likely important for host-parasite interactions and are potential targets for diagnosis. The host immune responses can be triggered by lectins, mucins and other enzymes [65–67], and can be modulated by parasite-derived miRNAs [36, 68, 69]. Extracellular vesicles excreted or secreted by helminths can alter host immune responses to parasite infection and clearance [36, 51]. Thus, apart from excretory/secretory (ES) products in the extracellular vesicles, parasite-derived miRNAs should also be taken into account in relation to parasite-host interactions, immune responses and the modulation thereof. In previous studies, miR-34, miR-71 and miR-100c were identified as common markers in the sera from hosts infected with *B. malayi*, *D. immitis*, *Litomosoides sigmodontis* and *L. loa* [51]. Considering the relative conservation of miR-34, miR-71 and miR-100c among some nematode species, we searched for these and other miRNAs found previously in dauer larvae of *C. elegans* [32], larvae of *A. suum* [52], extracellular vesicles of *H. polygyrus* [36] and *B. malayi* [51] and serum from hosts infected by *D. immitis*, *L. loa* or *O. ochengi* [49, 50, 70] among all *T. canis* miRNAs sequenced. Interestingly, the most prevalent, relatively conserved miRNAs, namely *Tc*-let-7-5p, *Tc*-lin-4, *Tc*-bantam, *Tc*-miR-10, *Tc*-miR-34, *Tc*-miR-71 and *Tc*-miR-100, were identified (Additional file 1: Table S8), suggesting key roles for them in modulating host/immunological responses. The common markers, such as let-7, bantam and miR-100, in parasitic nematodes might have implications for the diagnosis of infection or disease [37, 71–73]. In addition, interestingly, male-specifically regulated pathways were predicted as being linked to immune rather than reproductive processes; this aspect deserves future study in *T. canis*.

In addition to developmental regulation and immune modulation, some authors have proposed roles for miRNAs in drug resistance in pathogens [29, 38], and changes in transcription of potential drug targets, drug transporters, receptors and ion channels can associate with drug resistance [74]. Although mutations in P-glycoproteins [75], nicotinic acetylcholine receptors [76] and ligand-gated ion channels [77] have been reported to play important roles in anthelminthic resistance, no miRNAs identified in this study were inferred to target genes encoding such proteins. However, signalling processes and distinct biological functions in specific developmental stages are preferentially regulated by miRNAs (e.g., [78, 79]), and, in the present study, abundant miRNAs were predicted to regulate drug transport, metabolism and drug target pathways (Additional file 1: Table S9). Given the potential of miRNAs to target and suppress the expression of drug targets and host target genes, miRNA inhibitors or miRNA mimics might represent therapeutics to target parasite pathways and modulate parasite-host interactions [72, 74, 80].

## Conclusions

The present study of miRNAs in *T. canis* provides exciting prospects and delivers a resource to deepen and broaden our understanding of gene regulation in this enigmatic parasitic nematode. In particular, it provides a basis for experimental investigations of the developmental biology of the parasite, parasite-host interactions and disease, and might also assist in developing tools for the diagnosis of infection/disease, drug target discovery and drug resistance detection. Although this study focused on adult *T. canis*, the methods used should be readily applicable to different developmental stages and tissues of this and related parasites.

## Additional file

Additional file 1: Table S1. Primers for microRNA quantitative PCR. **Table S2.** Transcription profiles of known and novel microRNAs in *Toxocara canis*. **Table S3.** Gene ontology (GO) annotation and pathway enrichment analyses of microRNAs of *Toxocara canis*. **Table S4.** MicroRNAs differentially transcribed between male and female adults of *Toxocara canis*. **Table S5.** Sequence-dependent transcription between male and female adults of *Toxocara canis*. **Table S6.** GO annotation and pathway enrichment analysis of microRNAs differentially transcribed between male and female adults of *Toxocara canis*. **Table S7.** MicroRNA seed sequence families predicted to be involved in reproduction and larval development. **Table S8.** MicroRNAs predicted to be linked to host-parasite interactions. **Table S9.** MicroRNAs predicted to be drug targets or to have a link to drug resistance. (XLS 948 kb)

## References

[1] Macpherson CN (2013). The epidemiology and public health importance of toxocarosis: a zoonosis of global importance. Int J Parasitol..

[2] Nijsse R, Ploeger HW, Wagenaar JA, Mughini-Gras L (2015). *Toxocara canis* in household dogs: prevalence, risk factors and owners’ attitude towards deworming. Parasitol Res..

[3] Mendonça LR, Veiga RV, Dattoli VC, Figueiredo CA, Fiaccone R, Santos J (2012). *Toxocara* seropositivity, atopy and wheezing in children living in poor neighbourhoods in urban Latin American. PLoS Negl Trop Dis..

[4] Schoenardie ER, Scaini CJ, Brod CS, Pepe MS, Villela MM, McBride AJ (2013). Seroprevalence of *Toxocara* infection in children from southern Brazil. J Parasitol..

[5] Taira K, Saeed I, Permin A, Kapel CM (2004). Zoonotic risk of *Toxocara canis* infection through consumption of pig or poultry viscera. Vet Parasitol..

[6] Overgaauw PA, van Zutphen L, Hoek D, Yaya FO, Roelfsema J, Pinelli E (2009). Zoonotic parasites in fecal samples and fur from dogs and cats in the Netherlands. Vet Parasitol..

[7] Smith H, Holland C, Taylor M, Magnaval JF, Schantz P, Maizels R (2009). How common is human toxocarosis? Towards standardizing our knowledge. Trends Parasitol..

[8] EI-Tras WF, Holt HR, Tayel AA (2011). Risk of *Toxocara canis* eggs in stray and domestic dog hair in Egypt. Vet Parasitol..

[9] Despommier D (2003). Toxocarosis: clinical aspects, epidemiology, medical ecology, and molecular aspects. Clin Microbiol Rev..

[10] Caldera F, Burlone ME, Genchi C, Pirisi M, Bartoli E (2013). *Toxocara* encephalitis presenting with autonomous nervous system involvement. Infection..

[11] Kamuyu G, Bottomley C, Mageto J, Lowe B, Wilkins PP, Noh JC (2014). Exposure to multiple parasites is associated with the prevalence of active convulsive epilepsy in sub-Saharan Africa. PLoS Negl Trop Dis..

[12] Lee RM, Moore LB, Bottazzi ME, Hotez PJ (2014). Toxocarosis in North America: a systematic review. PLoS Negl Trop Dis..

[13] Li L, Gao W, Yang X, Wu D, Bi H, Zhang S (2014). Asthma and toxocarosis. Ann Allergy Asthma Immunol..

[14] Cassenote AJ, Lima AR, Pinto Neto JM, Rubinsky-Elefant G. Seroprevalence and modifiable risk factors for *Toxocara* spp. in Brazilian schoolchildren. PLoS Negl Trop Dis. 2014;8:e2830. 10.1371/journal.pntd.0002830 PMC4038482 24874504

[15] Cong W, Zhang XX, Zhou N, Yu CZ, Chen J, Wang XY (2014). *Toxocara* seroprevalence among clinically healthy individuals, pregnant women and psychiatric patients and associated risk factors in Shandong Province, Eastern China. PLoS Negl Trop Dis..

[16] Moreira GM, Telmo Pde L, Mendonça M, Moreira AN, McBride AJ, Scaini CJ (2014). Human toxocarosis: current advances in diagnostics, treatment, and interventions. Trends Parasitol..

[17] Chen J, Zhou DH, Nisbet AJ, Xu MJ, Huang SY, Li MW (2012). Advances in molecular identification, taxonomy, genetic variation and diagnosis of *Toxocara* spp. Infect Genet Evol..

[18] Holland CV, Smith HV (2006). *Toxocara* - The Enigmatic Parasite.

[19] Gasser RB (2013). A perfect time to harness advanced molecular technologies to explore the fundamental biology of *Toxocara* species. Vet Parasitol..

[20] Zhu XQ, Korhonen PK, Cai H, Young ND, Nejsum P, von Samson-Himmelstjerna G, et al. Genetic blueprint of the zoonotic pathogen *Toxocara canis*. Nat Commun. 2015;6:6145. 10.1038/ncomms7145 PMC4327413 25649139

[21] Shao C, Xu MJ, Alasaad S, Song HQ, Peng L, Tao JP, Zhu XQ (2014). Comparative analysis of microRNA profiles between adult *Ascaris lumbricoides* and *Ascaris suum*. BMC Vet Res..

[22] Bartel DP (2004). MicroRNAs: genomics, biogenesis, mechanism, and function. Cell..

[23] Grosshans H, Filipowicz W (2008). Molecular biology: the expanding world of small RNAs. Nature..

[24] Bartel DP (2009). MicroRNAs: target recognition and regulatory functions. Cell..

[25] Neeb ZT, Zahler AM (2014). An expanding world of small RNAs. Dev Cell..

[26] Lee RC, Feinbaum RL, Ambros V (1993). The *C. elegans* heterochronic gene *lin-4* encodes small RNAs with antisense complementarity to lin-14. Cell.

[27] Benfey PN (2003). Molecular biology: microRNA is here to stay. Nature..

[28] Schickel R, Boyerinas B, Park SM, Peter ME (2008). MicroRNAs: key players in the immune system, differentiation, tumorigenesis and cell death. Oncogene..

[29] Lalaouna D, Eyraud A, Chabelskaya S, Felden B, Massé E (2014). Regulatory RNAs involved in bacterial antibiotic resistance. PLoS Pathog..

[30] Bidarimath M, Khalaj K, Wessels JM, Tayade C (2014). MicroRNAs, immune cells and pregnancy. Cell Mol Immunol..

[31] Winter AD, Weir W, Hunt M, Berriman M, Gilleard JS, Devaney E (2012). Diversity in parasitic nematode genome: the microRNAs of *Brugia pahangi* and *Haemonchus contortus* are largely novel. BMC Genomics..

[32] Ahmed R, Chang Z, Younis AE, Langnick C, Li N, Chen W (2013). Conserved miRNAs are candidate post-transcriptional regulators of developmental arrest in free-living and parasitic nematodes. Genome Biol Evol..

[33] Cai P, Hou N, Piao X, Liu S, Liu H, Yang F (2011). Profiles of small non-coding RNAs in *Schistosoma japonicum* during development. PLoS Negl Trop Dis..

[34] Sun J, Wang S, Li C, Ren Y, Wang J (2014). Novel expression profiles of microRNAs suggest that specific miRNAs regulate gene expression for the sexual maturation of female *Schistosoma japonicum* after pairing. Parasit Vectors..

[35] Harris JF, Micheva-Viteva S, Li N, Hong-Geller E (2013). Small RNA-mediated regulation of host-pathogen interactions. Virulence..

[36] Buck AH, Coakley G, Simbari F, McSorley HJ, Quintana JF, Le Bihan T (2014). Exosomes secreted by nematode parasites transfer sRNAs to mammalian cells and modulate innate immunity. Nat Commun..

[37] Turner M, Galloway A, Vigorito E (2014). Noncoding RNA and its associated proteins as regulatory elements of the immune system. Nat Immunol..

[38] Devaney E, Winter AD, Britton C (2010). MicroRNAs: a role in drug resistance in parasitic nematodes?. Trends Parasitol..

[39] Jacobs DE, Zhu XQ, Gasser RB, Chilton NB (1997). PCR-based methods for identification of potentially zoonotic ascaridoid parasites of the dog, fox and cat. Acta Trop..

[40] Urquhart GM, Armour J, Duncan JL, Dunn AM, Jennings FW (2003). Veterinary Parasitology.

[41] http://soap.genomics.org.cn/ (2016). Accessed 13 Jan 2016.

[42] http://www.ncbi.nlm.nih.gov/ (2016). Accessed 13 Jan 2016.

[43] http://rfam.janelia.org/ (2016). Accessed 13 Jan 2016.

[44] http://www.mirbase.org/ (2016). Accessed 13 Jan 2016.

[45] http://sourceforge.net/projects/mireap/ (2016). Accessed 13 Jan 2016.

[46] Rehmsmeier M, Steffen P, Hochsmann M, Giegerich R (2004). Fast and effective prediction of microRNA/target duplexes. RNA..

[47] http://www.geneontology.org (2016). Accessed 13 Jan 2016.

[48] http://www.kegg.jp/kegg/pathway.html (2016). Accessed 13 Jan 2016.

[49] Tritten L, Burkman E, Moorhead A, Satti M, Geary J, Mackenzie C (2014). Detection of circulating parasite-derived microRNAs in filarial infections. PLoS Negl Trop Dis..

[50] Tritten L, O’Neill M, Nutting C, Wanji S, Njouendoui A, Fombad F (2014). *Loa loa* and *Onchocerca ochengi* miRNAs detected in host circulation. Mol Biochem Parasitol..

[51] Zamanian M, Fraser LM, Agbedanu PN, Harischandra H, Moorhead AR, Day TA (2015). Release of small RNA-containing exosome-like vesicles from the human filarial parasite *Brugia malayi*. PLoS Negl Trop Dis..

[52] Wang J, Czech B, Crunk A, Wallace A, Mitreva M, Hannon GJ, et al. Deep small RNA sequencing from the nematode *Ascaris* reveals conservation, functional diversification, and novel developmental profiles. Genome Res. 2011;21:1462–77. 10.1101/gr.121426.111 PMC3166831 21685128

[53] http://www.clustal.org/ (2016). Accessed 13 Jan 2016.

[54] Benes V, Castoldi M (2010). Expression profiling of microRNA using real-time quantitative PCR, how to use it and what is available. Methods..

[55] Iorio MV, Visone R, Di Leva G, Donati V, Petrocca F, Casalini P (2007). MicroRNA signatures in human ovarian cancer. Cancer Res..

[56] Pineles BL, Romero R, Montenegro D, Tarca AL, Han YM, Kim YM (2007). Distinct subsets of microRNAs are expressed differentially in the human placentas of patients with preeclampsia. Am J Obstet Gynecol..

[57] Shell S, Park SM, Radjabi AR, Schickel R, Kistner EO, Jewell DA (2007). Let-7 expression defines two differentiation stages of cancer. PNAS..

[58] Livak KJ, Schmittgen TD (2001). Analysis of relative gene expression data using real-time quantitative PCR and the 2(−Delta Delta C(T)) Method. Methods..

[59] Ameres SL, Zamore PD (2013). Diversifying microRNA sequence and function. Nat Rev Mol Cell Biol..

[60] Yates LA, Norbury CJ, Gilbert RJ (2013). The long and short of microRNA. Cell..

[61] Reinhart BJ, Slack FJ, Basson M, Pasquinelli AE, Bettinger JC, Rougvie AE (2000). The 21-nucleotide let-7 RNA regulates developmental timing in *Caenorhabditis elegans*. Nature..

[62] Hoogstrate SW, Volkers RJ, Sterken MG, Kammenga JE, Snoek LB (2014). Nematode endogenous small RNA pathways. Worm..

[63] Sarkies P, Selkirk ME, Jones JT, Blok V, Boothby T, Goldstein B (2015). ancient and novel small rna pathways compensate for the loss of piRNAs in multiple independent nematode lineages. PLoS Biol.

[64] Dieterich C, Sommer RJ (2009). How to become a parasite - lessons from the genomes of nematodes. Trends Genet..

[65] Theodoropoulos G, Hicks SJ, Corfield AP, Miller BG, Carrington SD (2001). The role of mucins in host-parasite interactions: Part II - helminth parasites. Trends Parasitol..

[66] Hewitson JP, Grainger JR, Maizels RM (2009). Helminth immunoregulation: the role of parasite secreted proteins in modulating host immunity. Mol Biochem Parasitol..

[67] Maizels RM (2013). *Toxocara canis*: molecular basis of immune recognition and evasion. Vet Parasitol..

[68] Zheng Y, Cai X, Bradley JE (2013). MicroRNAs in parasites and parasite infection. RNA Biol..

[69] Twu O, Johnson PJ (2014). Parasite extracellular vesicles: mediators of intercellular communication. PLoS Pathog..

[70] Hoy AM, Lundie RJ, Ivens A, Quintana JF, Nausch N, Forster T (2014). Parasite-derived microRNAs in host serum as novel biomarkers of helminth infection. PLoS Negl Trop Dis..

[71] O’Connell RM, Rao DS, Chaudhuri AA, Baltimore D (2010). Physiological and pathological roles for microRNAs in the immune system. Nat Rev Immunol..

[72] Manzano-Román R, Siles-Lucas M (2012). MicroRNAs in parasitic diseases: potential for diagnosis and targeting. Mol Biochem Parasitol..

[73] Marcilla A, Trelis M, Cortés A, Sotillo J, Cantalapiedra F, Minguez MT (2012). Extracellular vesicles from parasitic helminths contain specific excretory/secretory proteins and are internalized in intestinal host cells. PLoS One..

[74] Britton C, Winter AD, Gillan V, Devaney E (2014). MicroRNAs of parasitic helminths - Identification, characterization and potential as drug targets. Int J Parasitol Drugs Drug Resist..

[75] Prichard RK, Roulet A (2007). ABC transporters and beta tubulin in macrocyclic lactone resistance: prospects for marker development. Parasitology..

[76] Williamson SM, Robertson AP, Brown L, Williams T, Woods DJ, Martin RJ (2009). The nicotinic acetylcholine receptors of the parasitic nematode *Ascaris suum*: formation of two distinct drug targets by varying the relative expression levels of two subunits. PLoS Pathog..

[77] Rao VT, Siddiqui SZ, Prichard RK, Forrester SG (2009). A dopamine-gated ion channel (HcGGR3*) from *Haemonchus contortus* is expressed in the cervical papillae and is associated with macrocyclic lactone resistance. Mol Biochem Parasitol..

[78] Zhang L, Hammell M, Kudlow BA, Ambros V, Han M (2009). Systematic analysis of dynamic miRNA-target interactions during *C. elegans* development. Development.

[79] Leung AK, Sharp PA (2010). MicroRNA functions in stress responses. Mol. Cell..

[80] Schmidt MF (2014). Drug target miRNAs: chances and challenges. Trends Biotechnol..

